# Comparison of the level of cognitive processing between case-based items and non-case-based items on the Interuniversity Progress Test of Medicine in the Netherlands

**DOI:** 10.3352/jeehp.2018.15.28

**Published:** 2018-12-12

**Authors:** Dario Cecilio-Fernandes, Wouter Kerdijk, Andreas Johannes Bremers, Wytze Aalders, René Anton Tio

**Affiliations:** 1 Center for Education Development and Research in Health Professions (CEDAR), Research Group LEARN, University Medical Center Groningen, University of Groningen, Groningen, The Netherlands; 2 Department of Education and Research, Hanze University of Applied Sciences, Groningen, The Netherlands; 3 Department of Surgery, Radboud University Nijmegen Medical Center, Nijmegen, The Netherlands; 4 Department of Cardiology, Catharina Hospital, Eindhoven, The Netherlands; 5 Department of Educational Development and Research, Faculty of Health, Medicine and Life Sciences, Maastricht University, Maastricht, The Netherlands; Hallym University, Korea

**Keywords:** Educational assessment, Educational measurement, Medical education, Progress test, Netherlands

## Abstract

**Purpose:**

It is assumed that case-based questions require higher-order cognitive processing, whereas questions that are not case-based require lower-order cognitive processing. In this study, we investigated to what extent case-based and non-case-based questions followed this assumption based on Bloom’s taxonomy.

**Methods:**

In this article, 4,800 questions from the Interuniversity Progress Test of Medicine were classified based on whether they were case-based and on the level of Bloom’s taxonomy that they involved. Lower-order questions require students to remember or/and have a basic understanding of knowledge. Higher-order questions require students to apply, analyze, or/and evaluate. The phi coefficient was calculated to investigate the relationship between whether questions were case-based and the required level of cognitive processing.

**Results:**

Our results demonstrated that 98.1% of case-based questions required higher-level cognitive processing. Of the non-case-based questions, 33.7% required higher-level cognitive processing. The phi coefficient demonstrated a significant, but moderate correlation between the presence of a patient case in a question and its required level of cognitive processing (phi coefficient= 0.55, P< 0.001).

**Conclusion:**

Medical instructors should be aware of the association between item format (case-based versus non-case-based) and the cognitive processes they elicit in order to meet the desired balance in a test, taking the learning objectives and the test difficulty into account.

## Introduction

It is increasingly accepted that the purpose of assessments is not only to evaluate what has been learned but also to drive students’ learning [1,2]. However, assessments can only drive students’ learning when the learning objectives, test difficulty, and type of questions are aligned [3]. Although the educational aspect of assessments are imperative, ensuring the quality of items is also central, especially for high-stake tests. Since the Interuniversity Progress Test of Medicine in the Netherlands is a combination of a formative and summative assessment, both the educational aspect and the quality of the items should be aligned [4,5].

The Dutch progress test is a systematic longitudinal assessment that measures students’ knowledge at the level that they are expected to achieve by the end of their program [4,5]. On the Dutch progress test, students are assessed by case-based questions and non-case-based questions. Case-based questions better represent the type of reasoning students face in the clinical phase; thus, they are considered to be more authentic. Students, in theory, would rather answer case-based questions with a patient vignette than simple questions, because such questions are more closely related to their actual practice, similar to what they might encounter during their clinical rotations. Likewise, preclinical students correctly answered more questions that were not case-based, whereas clinical students correctly answered more case-based questions [6]. Although studies have suggested that case-based questions require students to reason about the case, while non-case-based questions only require students to recall certain pieces of knowledge, it is not clear whether these assumptions hold in a large-scale assessment in which many instructors are involved in writing items.

**Fig. 1.**
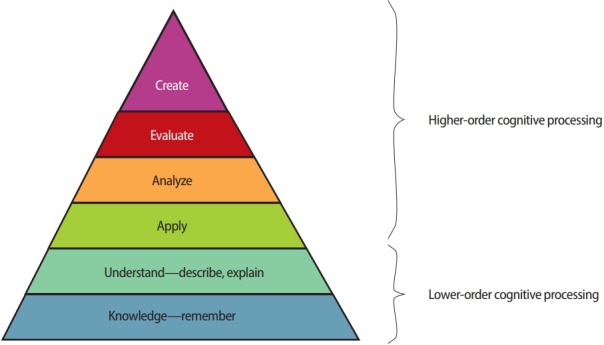
Levels of cognitive processing based on Bloom’s taxonomy.

A well-established framework to categorise questions is Bloom’s taxonomy, which provides a hierarchical model of knowledge acquisition that allows questions to be classified by the level of cognitive processing that they require. In theory, students achieve higher levels of cognitive processing only after mastering the lower levels of cognitive processing. The latter levels, in Bloom’s taxonomy, comprise knowledge reproduction and a minimal understanding of pieces of knowledge. The next level is application of information, followed by synthesising, evaluating, and creating information [7,8]. An illustration of this taxonomy is presented in Figure 1. Application of information may be considered as a transitional level of cognitive processing, or can be counted among the higher-order processes. In the current study, we assumed the latter.

Questions that require higher-order cognitive processing facilitate further knowledge consolidation, since students must recall their previous knowledge and then reason about that knowledge to make a decision. Questions that require higher-order cognitive processing have a greater impact on students’ knowledge retention. Studying with higher-order questions has show to improve students’ performance on lower- and higher-order questions when compared studying with lower-order questions [7].

Case-based questions thus require students to apply, synthesise, and evaluate their knowledge, assessing students’ ability to engage in higher-order cognitive processing. Non-case-based questions are expected to require students to remember and recall their knowledge, assessing students’ lower-order cognitive processing.

Instructors are often asked to produce case-based or non-case-based questions, with no specification of the appropriate cognitive level. One of the underlying assumptions of case-based questions is that they require higher-order cognitive processing, whereas questions that are not case-based usually require lower-order cognitive processing. In the current study, we investigated to what extent case-based and non-case-based questions followed this assumption based on Bloom’s taxonomy, since this may have implications for how instructors should formulate questions. More importantly, this may reflect the alignment between the objectives and the quality of the items of the Dutch progress test.

## Methods

### Ethical statement

For this study, we followed the Declaration of Helsinki and the privacy policy of the University of Groningen, and all data were anonymised and handled with confidentiality. Under Dutch law, no ethical approval was needed, because reanalysis of historical data is exempt.

### Study design

This is a retrospective study.

### Material

In this study, we analysed questions from the Dutch Interuniversity Progress Test of Medicine from 2007 to 2013. The progress test is a systematic, longitudinal assessment that aims to measure students’ knowledge at the level that they are expected to achieve by the end of their program, and the questions are based on the Dutch National Blueprint for the Medical Curriculum. The progress test is simultaneously administered 4 times a year to all undergraduate medical students, from the first to the sixth year. At the time of the study, 4 of the 8 medical schools in the Netherlands participated in the progress test. Each progress test consisted of 200 questions, which resulted in a total of 4,800 questions. This progress test is particularly suitable because each test contains 200 unique questions developed by a large and diverse group of instructors at different medical schools, it is independent of any curriculum, and it contains both case-based questions and non-case-based questions [4].

### Data analysis

Two of the authors (WA and RT) independently established for 50 questions whether a patient case was presented and what the required level of cognitive processing was according to Bloom’s taxonomy. Items were coded as case-based questions when a patient and illness were present. Items were coded as lower-order questions when items only required students to remember and/or show a basic understanding of knowledge, and when it was not necessary to use patient information to answer the question. Items were coded as higher-order questions when they required students to apply, analyse, or/and evaluate existing knowledge, and when students had to reason about the case to answer the question. The authors agreed on all questions about whether they were case-based, and there was only 1 disagreement about Bloom’s taxonomy. After discussing this question, the authors came to a consensus. Because of this high interrater agreement, the rest of the items were labelled by a single author (WA).

To study the relationship between the presence of a patient case and the required level of cognitive processing, the phi coefficient was calculated. The phi coefficient indicates the correlation between 2 binary variables [9]. The chi-square test was performed to compare the number of case-based questions that required higher-order cognitive processing with the number of non-case-based questions that required higher-order cognitive processing. Data were analysed using IBM SPSS ver. 21.0 (IBM Corp., Armonk, NY, USA).

## Results

Of the 4,800 questions, 1,138 were case-based (23.7%) and 3,662 were non-case-based. Regarding the level of cognitive processing that was required, 2,350 questions required higher-order cognitive processing and 2,450 required lower-order cognitive processing. There was a significant, but moderate association between the presence of a patient case in the question and its required level of cognitive processing (phi coefficient= 0.55, P< 0.001).

Of the 1,138 case-based questions, 1,116 required higher-level cognitive processing, while only 22 required lower-level cognitive processing. Of the 3,662 non-case-based questions, 1,234 required higher-level cognitive processing and 2,428 questions required lower-level cognitive processing (Table 1). This difference was significant (χ^2^(1)= 1,239.7, P< 0.001). The raw data are available in Supplement 1.

## Discussion

The current study investigated the relationship between whether a question was case-based and the level of cognitive processing, according to Bloom’s taxonomy, required to answer it. Case-based questions usually required higher-level cognitive processing, as expected. Interestingly, one-third of non-case-based questions also required higherlevel cognitive processing.

Our findings suggest that writing a case-based question causes instructors to produce questions that require a higher order of cognitive processing without explicitly asking them to do so. This may be because these questions are more closely related to real-life situations that are familiar to medical doctors in clinical practice. One might even argue that if a question is only aimed to measure recall or basic understanding, it may be redundant to include a patient case, since the reality of clinical practice usually involves dealing with a complex set of variables. If instructors find it challenging to produce higher-order questions, one might suggest that they use a relevant patient case as a basis for such questions.

Our findings also suggest that even without an explicit specification of the questions to be developed, more than one-third of the questions without a patient case required higher-order cognitive processing. Non-case-based questions can still require students to do more than just reproduce and show basic understanding. It is important to realise that the type of questions on a test should reflect the learning objectives and students’ level of development. Asking instructors to produce non-case-based questions and assuming that those questions will measure lower-order cognitive processing is unwarranted. Thus, it is imperative to provide guidance to instructors to write at the appropriate level of cognitive processing. If instructors write non-case-based questions, they must be instructed to write items requiring lower-level cognitive processing. Novice students will answer more lower-order than higher-order questions correctly, since they do not possess the necessary knowledge to handle cases that require knowledge application [6].

Because of the formative function and the repetitive character of the progress test [4,10], it is important that it include both questions that require lower-order cognitive processes and questions that require higher-order cognitive processing. The former question type may be desirable for novice students who have not yet acquired the necessary knowledge that they will eventually apply, and it may help them to develop the ability to apply their knowledge [11]. Thus, the use of questions that require lower- or higher-order cognitive processing should be aligned with the learning objectives and the test difficulty. Since the progress test is aimed at sixth-year students, it has been decided to expand the proportion of case-based questions on the progress test and to increase the level of complexity in the non-case-based items. Our findings suggest that the test may be too difficult for novices, since even the non-case-based questions require students to use higher-order cognitive processing. To better accommodate novice students, the use of a computerized adaptive test is under exploration.

Studies have investigated the content of questions on large-scale exams, such as the use of questions involving race/ethnicity [12] and the coverage and distribution of obesity-related questions [13]. However, little research has been done to assess the relationship between whether a question is case-based and the required level of cognitive processing on Bloom’s taxonomy in a question bank used for largescale assessments. Further investigation is required to explain the observed mismatch between non-case-based questions and higher-order cognitive processing. When categorizing the questions, we noticed that most of the questions related to ethics lacked patients, but assessed higher-order cognitive processing. Unfortunately, a thorough investigation was not conducted regarding this relationship across different disciplines, since discipline specificity was out of the scope of this study. Further research should investigate whether there are disciplines that are more liable to this mismatch than others. Identifying these disciplines may lead to a better alignment between the objectives and questions. Further research is also necessary to investigate the implications of using questions that require higher-order cognitive processing to test novices. In addition to requesting instructors to write case-based or non-case-based questions, they should be informed about the level of cognitive processing that is to be tested.

## Figures and Tables

**Table 1.** Distribution of case-based and non-case-based questions according to the required level of cognitive processing

	Case-based questions	Non-case-based questions
Lower-order cognitive processing	22 (1.9)	2,428 (36.3)
Higher-order cognitive processing	1,116 (98.1)	1,234 (33.7)

Values are presented as number (%).

## Supplementary materials

Supplement 1. Data files are available from https://doi.org/10.7910/DVN/U3TNY6

Supplement 2. Audio recording of the abstract.
